# Concentrations and Potential Health Risks of Metals in Lip Products

**DOI:** 10.1289/ehp.1205518

**Published:** 2013-05-02

**Authors:** Sa Liu, S. Katharine Hammond, Ann Rojas-Cheatham

**Affiliations:** 1 Environmental Health Sciences Division, School of Public Health, University of California, Berkeley, Berkeley, California, USA; 2 Asian Communities for Reproductive Justice,* Oakland, California, USA

**Keywords:** cosmetic safety, health risk, lipstick, metal, susceptible populations

## Abstract

Background: Metal content in lip products has been an issue of concern.

Objectives: We measured lead and eight other metals in a convenience sample of 32 lip products used by young Asian women in Oakland, California, and assessed potential health risks related to estimated intakes of these metals.

Methods: We analyzed lip products by inductively coupled plasma optical emission spectrometry and used previous estimates of lip product usage rates to determine daily oral intakes. We derived acceptable daily intakes (ADIs) based on information used to determine public health goals for exposure, and compared ADIs with estimated intakes to assess potential risks.

Results: Most of the tested lip products contained high concentrations of titanium and aluminum. All examined products had detectable manganese. Lead was detected in 24 products (75%), with an average concentration of 0.36 ± 0.39 ppm, including one sample with 1.32 ppm. When used at the estimated average daily rate, estimated intakes were > 20% of ADIs derived for aluminum, cadmium, chromium, and manganese. In addition, average daily use of 10 products tested would result in chromium intake exceeding our estimated ADI for chromium. For high rates of product use (above the 95th percentile), the percentages of samples with estimated metal intakes exceeding ADIs were 3% for aluminum, 68% for chromium, and 22% for manganese. Estimated intakes of lead were < 20% of ADIs for average and high use.

Conclusions: Cosmetics safety should be assessed not only by the presence of hazardous contents, but also by comparing estimated exposures with health-based standards. In addition to lead, metals such as aluminum, cadmium, chromium, and manganese require further investigation.

Cosmetic products contain thousands of chemicals, some of which have been associated with reproductive, developmental, or other health effects based on human or animal studies, including phthalates, formaldehyde, methylene chloride, acetone, acetonitrile, methacrylates, toluene, xylene, ethyl ether, and lead (Pb) (De Cock et al. 2012; Dodson et al. 2012; Ferret et al. 2012; Heisterberg 2012; Lefebvre et al. 2012; Moyer and Nixon 2012; Ulker et al. 2012). Lip products have been suggested as a particular concern because of the potential for exposure through ingestion (Loretz et al. 2005).

In October 2007, the Campaign for Safe Cosmetics tested 33 popular brands of lipsticks and reported that 61% contained Pb, with levels up to 0.65 parts per million, indicating a cause for concern (Campaign for Safe Cosmetics 2007). Although the Campaign for Safe Cosmetics report was not peer reviewed, it brought attention to the issue of chemicals in cosmetic and personal care products and their safety. Since then, two other studies evaluated Pb in eye shadows and lipsticks: a U.S. Food and Drug Administration (FDA) study that detected Pb in all tested lipsticks (Hepp et al. 2009) and a study (Al-Saleh et al. 2009) that identified several cosmetic products containing Pb > 20 ppm, the FDA limit of Pb as an impurity in color additives for cosmetics (FDA 2011). Studies conducted in other countries have also detected Pb and cadmium (Cd) in some lipstick samples (Adepoju-Bello et al. 2012; Brandao et al. 2012; Gondal et al. 2010; Solidum and Peji 2011).

In the present study we extended the FDA study and the Campaign for Safe Cosmetics by testing for Pb, aluminum (Al), Cd, cobalt (Co), chromium (Cr), copper (Cu), manganese (Mn), nickel (Ni), and titanium (Ti) in lipsticks and lip glosses used by young women, estimating potential daily intakes, and comparing the estimates to existing health guidelines.

## Method

*Sample collection*. A convenience sample of lipsticks and lip glosses was selected based on information provided by 12 members of the Asian Communities for Reproductive Justice (ACRJ) Youth Program, a group of Asian girls 14–19 years of age who lived in low-income neighborhoods in Oakland, California. Specifically, the girls were asked to record the brand and product names of the lipsticks and lip glosses they were carrying and had in their bathrooms at home, which represented products used by their sisters as well. The reported products were then purchased by researchers at a chain drug store (26 products), a major department store (4 products), and a chain specialty store (2 products).

All methods were approved by the University of California, Berkeley, Institutional Review Board (IRB). The young women who provided information on the lip products they used, and their parents or guardians, signed an informed consent form approved by the University of California, Berkeley, IRB before the study. We have complied with all applicable IRB regulations and requirements.

*Analytical method*. Sample analysis followed National Institute for Occupational Safety and Health (NIOSH) standard method for metals (Method 7303: Hot Block/HCl/HNO3 Digestion) with slight modifications (NIOSH 2003). Approximately 0.5 g of each sample was transferred into a clean, 50-mL hot block digestion tube and digested with 2.0 mL concentrated nitric acid (HNO_3_) on a block digester (LACHAT Instruments, Loveland, CO) at 130°C for 15 hr, with the tubes covered with glass funnels to allow for nitric acid reflux during the digestion. Samples were diluted to 12.5 mL with distilled, deionized water and then filtered to remove material that did not completely dissolve, including waxy material floating on the top of the digest and white or light yellow precipitates that were most likely silicates. Solutions were analyzed by inductively coupled plasma optical emission spectrometry (ICP/OES) (Optima 5300DV; PerkinElmer, Waltham, MA). Metals examined included Al, Cd, Co, Cr, Cu, Mn, Pb, Ni, and Ti. Reagent blanks and media blanks were also analyzed.

*Data analysis*. Measured metal concentrations (parts per million, weight/weight) were converted to estimated daily metal intakes (micrograms per day) based on lip product use data from a study of cosmetic product use among 360 women (ages 19–65 years) from ten different U.S. geographical regions (Loretz et al. 2005). The investigators reported that on average the women used lipsticks 2.35 times per day (range, 0–20 times) and applied 10 mg of product at each use (range, 0–214 mg), resulting in average daily use of 24 mg of lip products (range, 0–214 mg; 95th percentile = 87 mg/day). We assumed that all applied lip products were ingested, and thus estimated metal intakes for average use (24 mg/day) and high use (87 mg/day) of lip products.

Metals in cosmetic products are not currently regulated in the United States. Therefore, as a point of comparison for potential health risks, we estimated acceptable daily intakes (ADIs) for Al, Cd, Cu, Ni, and Pb based on information used by the California Environmental Protection Agency (Cal/EPA) to determine Public Health Goals (PHGs) for drinking water (Cal/EPA 2001a, 2001b, 2006, 2008b, 2009) (Table 1). Specifically, we derived ADIs based on the following no observed adverse effect levels (NOAELs) or lowest observed adverse effect levels (LOAELs) and uncertainty factors (UF) used to determine PHGs: Al NOAEL/LOAEL = 125 mg/day and UF = 100 (10 for duration of study, 10 for interindividual variation and sensitive subgroups) (Cal/EPA 2001a); Cd NOAEL = 19 μg/day and UF = 50 (5 for protecting sensitive individuals, 10 for cancer risk due to oral exposure to Cd) (Cal/EPA 2006); Cu NOAEL = 426 μg/kg-day and UF = 3 for uncertainties in study data (Cal/EPA 2008b); Ni NOAEL = 1.12 mg/kg-day and UF = 1,000 (10 for interspecies extrapolation, 10 for intraspecies variability, and 10 for potential carcinogenicity of oral exposure) (Cal/EPA 2001b); Pb NOAEL/LOAEL = 2.86 μg/day and UF = 3 for uncertainty in protectiveness of this level and small sample size (Cal/EPA 2009). For NOAEL/LOAEL reported according to body weight per day (micrograms per kilogram per day) we assumed a body weight of 50 kg for young Asian women to determine the ADI.

**Table 1:** PHGs and ADI^*a*^ derived for the present study.

Metal intake guidelines	Al	Cd	Cr^*b*^	Cu	Mn^*c*^	Ni	Pb
PHG^*d*^ (μg/L)	600	0.04	0.02	300	0.09	12	0.2
NOAEL/LOAEL	125 mg/day	19 μg/day	0.5/mg/kg-day	426 μg/kg-day	NA	1.12 mg/kg-day	2.86 μg/day
UF	100	50	NA	3	NA	1,000	3
ADI (μg/day)	1,250	0.38	0.1	7,100	1.8	56	0.95
Abbreviations: ADI, acceptable daily intake; NA, not applicable; PHGs, public health goals; UF, uncertainty factor. ^***a***^See “Methods” for the calculation of ADI.^***b ***^For PHG, Cr(VI) potency factor was used instead of NOAEL/LOAEL.^***c***^REL for manganese via inhalation was used instead of a PHG value (μg/m^3^).^***d***^As reported in Cal/EPA PHG documents (Cal/EPA 2001a, 2001b, 2006, 2008a, 2008b, 2009, 2011b).							

Our ADI for Cr was based on the PHG derived by the Cal/EPA for carcinogenic risks associated with hexavalent Cr according to the standard risk calculation [concentration = risk/(potency × dose)] (Cal/EPA 2011b), such that ADI = risk/*P*_o_, where risk = a default risk level of 1 in 1 million, or 10^–6^, and *P*_o_ = 0.5/mg/kg-day, the oral cancer potency for hexavalent Cr, resulting in an estimated ADI of 0.1 µg/day for a 50-kg woman. Mn does not have a PHG, so we used the California Reference Exposure Level (REL) for systemic effects of Mn via inhalation of 20 m^3^ of air per day (Cal/EPA 2008a), assuming that toxicokinetic differences between oral and inhalational routes of exposure were not significant. Co and Ti have no PHGs or RELs because they are not regulated by California or federal standards; therefore we did not derive ADIs for these metals.

Finally, we compared estimated metal intakes via lip products to the derived acceptable daily intakes. We derived relative intake indices (RIIs) for metals via lip products as a percentage of the ADI:

relative intake index (RII) % = (estimated daily intake/ADI) × 100%, [1]

Hence, for each metal, intake at the ADI would yield an RII of 100%. RIIs were calculated assuming average use of lip products (intake of 24 mg of product/day) and high use (87 mg/day).

## Results

*Lip product information*. We tested 32 individual products in this preliminary study, including 8 lipsticks and 24 lip glosses sold by a total of 7 distinct companies. Prices ranged from $5.59 to $24. The tested products were representative of those used by young women in the ACRJ Youth Program.

**Figure 1:**
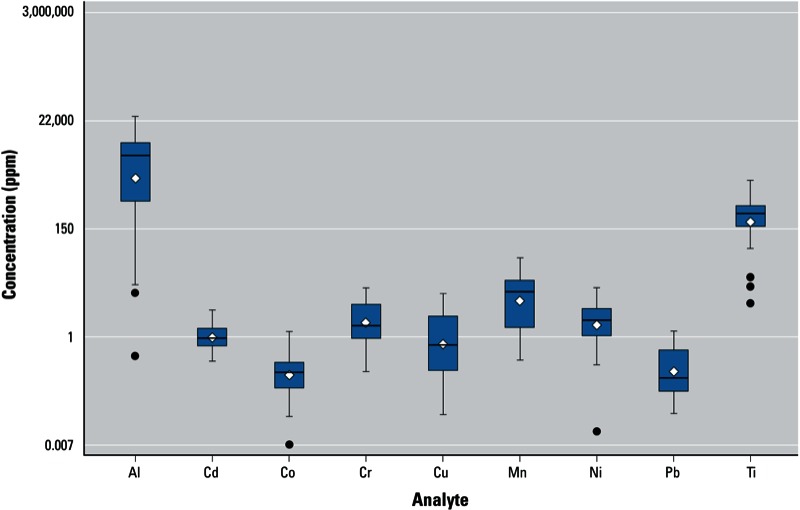
Box and whiskers plot showing the distributions of the measured concentrations for each metal. Corresponding numeric data are provided in Table 2 for all samples (*n* = 32). Boxes extend from the 25th to the 75th percentile, horizontal bars inside the boxes represent the median, diamonds represent the mean, whiskers extend to maximum and minimum observations within 1.5 times the length of the interquartile range above and below the 75th and 25th percentiles, respectively, and outliers are represented as circles.

*Metal concentrations in lip products*. Mn, Ti, and Al were detected in all examined products, with Ti and Al present in the highest concentrations of the metals tested (Table 2 and Figure 1). Pb was detected in 75% of products, with an average concentration of 0.36 ± 0.39 ppm (median, 0.151 ppm; maximum, 1.32 ppm). Approximately half (47%) of the samples contained Pb at concentrations higher than the FDA-recommended maximum level of 0.1 ppm for Pb in candy likely to be consumed frequently by small children (FDA 2005). Co had the lowest average concentration among the examined metals (0.28 ± 0.31 ppm, mean ± SD). Metal concentrations varied substantially across the products (Table 2). For example, product L1014 had the highest Cr concentration (9.72 ppm) and the second highest concentrations of Cd, Mn, and Pb (2.16, 35.3, and 1.25 ppm, respectively). Products L1021 and L1029 had the highest concentrations of Pb (1.32 ppm) and Al (27,032 ppm), respectively, and both had high Cr and Mn levels. However, we did not observe clear patterns indicating that metal concentrations were related to specific brands, product type (lipstick vs. lip gloss), color, or cost.

**Table 2:** Metal concentration in tested lip products and summary statistics (ppm w/w).

Sample ID	Type	Al	Cd	Co	Cr	Cu	Mn	Ni	Pb	Ti
L1001	Lip gloss	2,147	< 0.002	0.133	0.584	1.19	3.35	2.10	0.077	135
L1002	Lip gloss	4,413	0.667	0.897	4.19	2.05	29.5	4.23	0.405	663
L1003	Lip gloss	4,559	< 0.002	0.302	1.32	0.579	5.39	9.14	0.149	265
L1004	Lip gloss	520	3.48	0.253	0.697	0.889	0.884	9.73	< 0.025	214
L1005	Lip gloss	164	1.63	< 0.005	0.386	0.689	0.700	3.59	0.080	329
L1006	Lip gloss	10,536	< 0.002	0.200	1.21	0.319	6.83	0.651	0.097	454
L1007	Lip gloss	547	0.333	0.092	0.205	1.19	1.64	0.397	0.042	103
L1008	Lip gloss	10,533	< 0.002	0.304	1.20	1.03	6.78	1.85	< 0.025	958
L1009	Lip gloss	4,079	0.953	0.961	4.94	0.197	38.5	2.71	0.572	1,418
L1010	Lip gloss	1,078	< 0.002	0.161	6.05	0.534	1.48	2.98	< 0.025	369
L1011	Lip gloss	0.415	1.07	0.059	< 0.005	0.063	0.35	0.013	0.082	4.72
L1012	Lip gloss	1,701	< 0.002	0.176	0.799	0.125	3.20	3.27	< 0.025	278
L1013	Lip gloss	547	< 0.002	0.141	1.28	< 0.010	10.2	0.299	0.216	60.0
L1014	Lipstick	4,448	2.16	1.30	9.72	< 0.010	35.3	3.02	1.25	399
L1015	Lipstick	10,730	0.479	0.025	3.27	< 0.010	13.3	3.61	< 0.025	895
L1016	Lipstick	11,682	0.694	0.106	3.90	< 0.010	23.3	1.41	0.128	563
L1017	Lip gloss	306	< 0.002	0.099	0.648	0.256	0.597	0.51	0.050	262
L1018	Lip gloss	5,815	< 0.002	0.218	3.18	4.21	11.3	4.32	0.079	368
L1019	Lip gloss	3,314	< 0.002	0.214	5.06	6.81	10.0	4.57	1.04	247
L1020	Lip gloss	5,986	< 0.002	0.243	2.05	0.492	8.91	3.48	< 0.025	346
L1021	Lip gloss	4,448	0.962	0.652	7.84	5.71	28.6	6.27	1.32	460
L1022	Lip gloss	9,625	< 0.002	0.199	4.37	7.35	11.0	3.66	0.421	307
L1023	Lip gloss	5,007	< 0.002	0.332	4.42	7.38	14.0	4.66	0.710	973
L1024	Lip gloss	11.4	1.26	0.066	1.39	2.58	0.661	1.69	0.519	15.9
L1025	Lip gloss	7.72	0.896	0.007	0.326	0.125	0.510	0.278	0.029	10.1
L1026	Lipstick	10,585	< 0.002	< 0.005	0.948	0.028	8.22	1.08	0.133	294
L1027	Lipstick	11,131	1.46	0.19	6.53	< 0.010	21.5	1.85	0.678	304
L1028	Lip gloss	3,911	< 0.002	0.074	1.20	< 0.010	8.16	1.25	0.153	125
L1029	Lipstick	27,032	0.908	0.381	7.03	< 0.010	35.1	2.17	< 0.025	328
L1030	Lipstick	6,369	< 0.002	< 0.005	1.36	< 0.010	8.39	0.673	0.296	303
L1031	Lip gloss	5,511	< 0.002	< 0.005	3.27	0.214	6.35	1.63	< 0.025	228
L1032	Lipstick	14.2	0.426	0.058	< 0.005	< 0.010	0.361	< 0.012	0.0997	4.64
All samples
LOD		0.025	0.002	0.005	0.005	0.01	0.002	0.012	0.025	0.01
Maximum		27,032	3.48	1.30	9.72	7.38	38.5	9.73	1.32	1,418
Minimum		0.415	< 0.002	< 0.005	< 0.005	< 0.010	0.350	< 0.012	< 0.025	4.64
Percent > LOD		100	47	88	94	72	100	97	75	100
For values > LOD										
Median		4,431	0.953	0.194	1.72	0.689	8.19	2.17	0.151	303
Mean		5,211	1.16	0.28	2.98	1.91	11.1	2.81	0.359	365
SD		5,570	0.805	0.307	2.56	2.51	11.4	2.36	0.387	318
LOD, limit of detection.										

**Figure 2:**
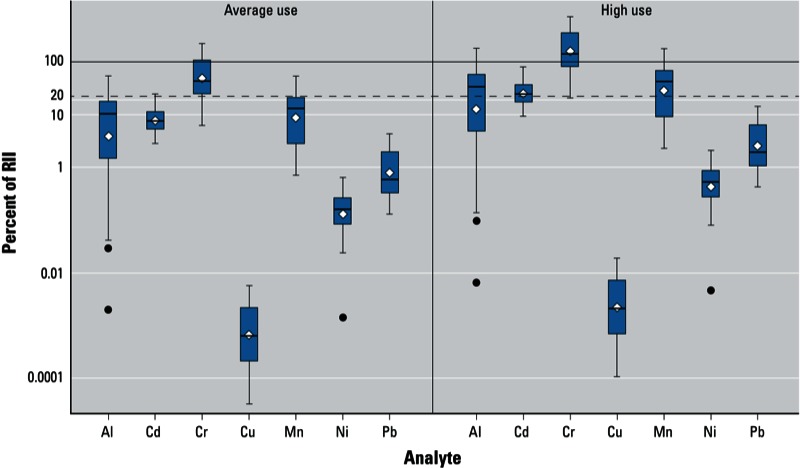
Box and whiskers plots showing distributions of RII values for each metal assuming average use or high use of lip products, defined as 24 and 87 mg of product/day, respectively. Corresponding numeric data are provided in Table 3 for all samples (*n* = 32). Boxes extend from the 25th to the 75th percentile, horizontal bars inside the boxes represent the median, diamonds represent the mean, whiskers extend to maximum and minimum observations within 1.5 times the length of the interquartile range above and below the 75th and 25th percentiles, respectively, and outliers are represented as circles. RII values represent the estimated daily intake for each metal as a percentage of the ADI values derived for this study. The horizontal line at RII = 100 indicates daily intakes that are equal to the ADI values for each metal; the horizontal line at RII = 20 indicates estimated daily intakes that are 20% of the ADI.

*Estimated daily intakes via use of lip products*. We converted measured metal concentrations (parts per million) in the individual lip product samples to metal intakes (micrograms per day) based on usage patterns reported by Loretz et al. (2005), assuming average and high use (resulting in oral intake of 24 and 87 mg of product per day, respectively) (Table 3). RIIs comparing estimated metal intakes from lip products to the derived acceptable daily intake are presented in Figure 2. When used at the average daily rate (24 mg/day), estimated Cr intake from 10 products (31%) exceeded the ADI for Cr (RII > 100%). Estimates based on high use (87 mg/day) suggested exposures exceeding the ADI for Al in 1 sample (3% of the products tested), Cr in 22 samples (68%), and Mn in 7 samples (22%). Estimated intakes for Ni, Cu, and Pb were well below their ADIs even for high use. Estimated Pb intake for the product with the highest Pb concentration (product L1021) was 3% and 12% of the ADI assuming average and high use, respectively.

**Table 3:** Estimated metal intakes via lip products for average (24 mg/day) and high (87 mg/day) daily use (μg/day).

Sample ID	Al		Cd		Co		Cr		Cu		Mn		Ni		Pb		Ti	
	Average	High	Average	High	Average	High	Average	High	Average	High	Average	High	Average	High	Average	High	Average	High
L1001	52	187	< 0.00005	< 0.000174	0.0032	0.012	0.014	0.051	0.028	0.10	0.08	0.29	0.050	0.18	0.0018	0.0067	3.2	12
L1002	106	384	0.016	0.058	0.022	0.078	0.10	0.36	0.049	0.18	0.71	2.6	0.10	0.37	0.010	0.035	16	58
L1003	109	397	< 0.00005	< 0.00017	0.0072	0.026	0.032	0.12	0.014	0.050	0.13	0.47	0.22	0.80	0.0036	0.013	6.4	23
L1004	12	45	0.084	0.30	0.0061	0.022	0.017	0.061	0.021	0.077	0.021	0.077	0.23	0.85	< 0.0006	< 0.0022	5.1	19
L1005	4	14	0.039	0.14	< 0.0001	< 0.0004	0.0093	0.034	0.017	0.060	0.017	0.061	0.086	0.31	0.0019	0.0069	7.9	29
L1006	253	917	< 0.00005	< 0.00017	0.0048	0.017	0.029	0.11	0.0077	0.028	0.16	0.59	0.016	0.06	0.0023	0.0084	11	40
L1007	13	48	0.008	0.029	0.0022	0.0080	0.0049	0.018	0.029	0.10	0.039	0.14	0.010	0.03	0.0010	0.0037	2.5	9.0
L1008	253	916	< 0.00005	< 0.00017	0.0073	0.026	0.029	0.10	0.025	0.089	0.16	0.59	0.044	0.16	< 0.0006	< 0.0022	23	83
L1009	98	355	0.023	0.083	0.023	0.084	0.12	0.43	0.0047	0.017	0.92	3.4	0.065	0.24	0.014	0.050	34	123
L1010	26	94	< 0.00005	< 0.00017	0.0039	0.014	0.15	0.53	0.013	0.046	0.035	0.13	0.072	0.26	< 0.0006	< 0.0022	8.9	32
L1011	0.010	0.036	0.026	0.093	0.0014	0.0051	< 0.0001	< 0.0004	0.0015	0.005	0.0084	0.030	0.00031	0.0011	0.0020	0.0072	0.1	0.41
L1012	41	148	< 0.00005	< 0.00017	0.0042	0.015	0.019	0.070	0.0030	0.011	0.077	0.28	0.079	0.28	< 0.0006	< 0.0022	6.7	24
L1013	13	48	< 0.00005	< 0.00017	0.0034	0.012	0.031	0.11	< 0.0002	< 0.0009	0.25	0.89	0.0072	0.026	0.0052	0.019	1.4	5.2
L1014	107	387	0.052	0.19	0.031	0.11	0.23	0.85	< 0.0002	< 0.0009	0.85	3.1	0.072	0.26	0.030	0.11	9.6	35
L1015	258	933	0.012	0.042	0.00061	0.0022	0.079	0.28	< 0.0002	< 0.0009	0.32	1.2	0.087	0.31	< 0.0006	< 0.0022	21	78
L1016	280	1,016	0.017	0.060	0.0025	0.0092	0.094	0.34	< 0.0002	< 0.0009	0.56	2.0	0.034	0.12	0.0031	0.011	13.5	49
L1017	7.3	27	< 0.00005	< 0.00017	0.0024	0.0086	0.016	0.060	0.0061	0.022	0.014	0.052	0.012	0.044	0.0012	0.0044	6.3	23
L1018	140	506	< 0.00005	< 0.00017	0.0052	0.019	0.076	0.28	0.10	0.37	0.27	0.99	0.10	0.38	0.0019	0.0069	8.8	32
L1019	80	288	< 0.00005	< 0.00017	0.0051	0.019	0.12	0.44	0.16	0.59	0.24	0.87	0.11	0.40	0.025	0.090	5.9	21
L1020	144	521	< 0.00005	< 0.00017	0.0058	0.021	0.049	0.18	0.012	0.04	0.21	0.77	0.084	0.30	< 0.0006	< 0.0022	8.3	30
L1021	107	387	0.023	0.084	0.016	0.057	0.19	0.68	0.14	0.50	0.69	2.5	0.15	0.55	0.032	0.11	11	40
L1022	231	837	< 0.00005	< 0.00017	0.0048	0.017	0.10	0.38	0.18	0.64	0.26	0.96	0.088	0.32	0.010	0.037	7.4	27
L1023	120	436	< 0.00005	< 0.00017	0.0080	0.029	0.11	0.38	0.18	0.64	0.33	1.2	0.11	0.41	0.017	0.062	23	85
L1024	0.27	0.99	0.030	0.11	0.0016	0.0057	0.033	0.12	0.062	0.22	0.016	0.058	0.040	0.15	0.012	0.045	0.38	1.4
L1025	0.19	0.67	0.022	0.078	0.00017	0.00061	0.0078	0.028	0.003	0.011	0.012	0.044	0.0067	0.024	0.00070	0.0025	0.24	0.88
L1026	254	921	< 0.00005	< 0.00017	< 0.0001	< 0.0004	0.023	0.082	0.00067	0.0024	0.20	0.72	0.026	0.094	0.0032	0.012	7.1	26
L1027	267	968	0.035	0.13	0.0046	0.017	0.16	0.57	< 0.0002	< 0.0009	0.52	1.9	0.044	0.16	0.016	0.059	7.3	26
L1028	93.9	340	< 0.00005	< 0.00017	0.0018	0.0064	0.029	0.10	< 0.0002	< 0.0009	0.20	0.71	0.030	0.11	0.0037	0.013	3.0	11
L1029	649	2,352	0.022	0.079	0.0091	0.033	0.17	0.61	< 0.0002	< 0.0009	0.84	3.1	0.052	0.19	< 0.0006	< 0.0022	7.9	29
L1030	153	554	< 0.00005	< 0.00017	< 0.0001	< 0.0004	0.033	0.12	< 0.0002	< 0.0009	0.20	0.73	0.016	0.059	0.0071	0.026	7.3	26
L1031	132	479	< 0.00005	< 0.00017	< 0.0001	< 0.0004	0.079	0.28	0.0051	0.019	0.15	0.55	0.039	0.14	< 0.0006	< 0.0022	5.5	20
L1032	0.34	1.2	0.010	0.037	0.0014	0.0051	< 0.0001	< 0.0004	< 0.0002	< 0.0009	0.0087	0.031	< 0.0003	< 0.0010	0.0024	0.0087	0.11	0.40
All samples
Maximum	649	2,352	0.084	0.30	0.031	0.11	0.23	0.85	0.18	0.64	0.92	3.4	0.23	0.85	0.030	0.11	34	123
Minimum	0.010	0.036	< 0.00005	< 0.00017	< 0.0001	< 0.0004	< 0.0001	< 0.0004	< 0.0002	< 0.0009	0.0084	0.030	< 0.0003	< 0.0010	< 0.0006	< 0.0022	0.11	0.40
For values > LOD
Median	106	385	0.023	0.083	0.0047	0.017	0.041	0.15	0.017	0.060	0.20	0.71	0.052	0.19	0.0036	0.013	7.3	26
Mean	125	453	0.028	0.10	0.0067	0.024	0.072	0.26	0.046	0.17	0.27	0.96	0.067	0.24	0.0086	0.031	8.8	32
SD	134	485	0.019	0.070	0.0074	0.027	0.061	0.22	0.060	0.22	0.27	0.99	0.057	0.21	0.0093	0.034	7.6	28
LOD, limit of detection.																		

## Discussion

This preliminary study of metal content in lip products suggests potential public health concerns. However, metals in cosmetic products are not currently regulated by the FDA. Although metal concentrations in lip products have been reported by studies both in the United States and in other countries (Adepoju-Bello et al. 2012; Al-Saleh and Al-Enazi 2011; Al-Saleh et al. 2009; Brandao et al. 2012; Gondal et al. 2010; Gunduz and Akman 2013; Hepp et al. 2009; Solidum and Peji 2011), interpreting how reported concentrations may be related to potential health risk is challenging. We used California Public Health Goals for drinking water contaminants to derive health-based standards for ingestion exposure from drinking water (Cal/EPA 2011a). In the calculation of PHG, a relative source contribution is applied to adjust intake of the contaminant from sources other than drinking water. Vulnerable populations such as infants and children are also considered by adjusting water consumption rate (dose) for the different age groups. PHGs provide information on concentrations of drinking-water contaminants that pose no significant health risks if the water is consumed for a lifetime. Although they are not regulatory standards, PHGs are considered more health based than Maximum Contaminant Levels, which are mandatory drinking-water standards that take into account not only health risks but also the feasibility and cost of monitoring and maintaining standards in drinking-water supplies. In deriving the ADIs we did not account for metal intakes from other sources, nor did we consider potential age- and sex-related vulnerabilities, although they may have been partially accounted for by the uncertainty factors used in deriving PHGs. We used 20% of RII as an additional comparison point because 20% is a typical relative source contribution value used in developing PHGs for the tested metals. We found that estimated intakes of Al, Cd, Cr, and Mn from some of the tested products were > 20% of their estimated ADIs, assuming average daily use of lip products. The proportion of samples with RII > 20% substantially increased assuming high use (corresponding to the 95th percentile of lip product use based on a previous study) (Loretz et al. 2005), including 63% of the products tested for Al, 31% for Cd, 91% for Cr, and 66% for Mn.

Cd and its compounds are known human carcinogens [International Agency for Research on Cancer (IARC) 1993]. Inhalation exposure of Cd has been associated with lung cancer and respiratory system damage (Chan et al. 1988; Davison et al. 1988; Nawrot et al. 2006; Smith et al. 1976; Stayner et al. 1992; Thun et al. 1985), and chronic oral exposure may lead to kidney and bone impairments (Åkesson et al. 2005; Nogawa et al. 1990). Animal studies indicate that young animals might absorb more Cd than adults and be more susceptible to bone impairments (Ogoshi et al. 1989). Animal studies also found that feeding rats and mice high level of Cd (1–20 mg/kg-day) during pregnancy resulted in low birth weight, affected skeleton development, and behavior and learning problems [Agency for Toxic Substances and Disease Registry (ATSDR) 2008a]. Although less than half (47%) of the tested lip products had detectable levels of Cd, our results suggest that Cd intake could exceed 20% of our estimated ADI for Cd exposure via drinking water for one product assuming average use, and for 10 products assuming high use.

Cr(VI) is a known human carcinogen; inhalation causes lung cancer and oral exposure through drinking water has been linked with increased stomach tumors (ATSDR 2008b). Our measurements did not distinguish hexavalent Cr from less toxic forms, and the percentage of hexavalent Cr in the lip products is therefore unknown. However, high total estimated intakes of Cr from use of several lip products and the potential for additional exposure from other sources suggests that Cr intake from lip products should be a priority for additional research.

Our acceptable daily intake value for Mn was derived from the Cal/EPA reference exposure limit for inhalational exposure because a public health goal is not available for Mn in drinking water. Inhalational exposure to high levels of Mn in occupational settings causes neurological effects in humans (ATSDR 2008c; Cook et al. 1974; Crossgrove and Zheng 2004). Although the evidence is inconclusive, Mn in drinking water has been associated with neurological and neurobehavioral outcomes in children, which suggests that effects of oral exposure may be similar to effects associated with inhalational exposure (Bouchard et al. 2006; Kilburn 1987; Kondakis et al. 1989). Estimated Mn intake assuming high use of lip products exceeded our ADI value based on inhalational exposure for seven products (22% of tested products).

Although Pb was detected in 75% of the lip product samples, including 15 samples with concentrations higher than the FDA standard of 0.1 ppm for Pb in candy frequently consumed by children, RIIs for estimated Pb intakes appeared to be low compared with RIIs for Al, Cd, Cr, and Mn. Thus, although Pb in lip products has been intensively discussed (Al-Saleh and Al-Enazi 2011; Al-Saleh et al. 2009; Bach and Newman 2010), other metals in the lip products should also be investigated. Nevertheless, it is generally accepted that there is no safe level of Pb intake (Centers for Disease Control and Prevention 2012), and the federal maximum contaminant level goal for Pb in drinking water is zero (U.S. Environmental Protection Agency 2012). The European Union Cosmetics Directive lists Cd, Cr, and Pb and their compounds as unacceptable constituents of cosmetic products (Salvador and Chisvert 2007).

The digestion method used in this study did not completely dissolve the lip product samples. The recent FDA study, which used a more complete digestion method to determine the total Pb concentration in lipsticks, reported an average Pb content of 1.07 ppm (range, 0.09–3.06 ppm) in 22 tested lipsticks (Hepp et al. 2009), in contrast with an average Pb concentration of 0.36 ppm (range, < 0.025–1.32 ppm) for our sample of 32 lip products. Differences between the studies may reflect variation in Pb content among the specific products tested, though incomplete digestion of our samples also may have reduced apparent concentrations relative to actual levels. Therefore, future studies should endeavor to measure total metal content to the degree possible.

## Conclusion

Our data indicate the need for further studies to evaluate metal concentrations in lip products, as well as other cosmetics, and related potential health risks. In addition to Pb, metals such as Al, Cd, Cr, and Mn require further investigation. Cosmetics safety should be assessed not only by the presence of hazardous contents, but also by comparing estimated exposures with health based standards. This preliminary study of the metal content of 32 lip products suggests that toxic metals in cosmetics should be regulated to protect women’s health in the United States, as has already been undertaken by the European Union through their Cosmetics Directive.

## References

[r1] Adepoju-Bello AA, Oguntibeju OO, Adebisi RA, Okpala N, Coker HAB (2012). Evaluation of the concentration of toxic metals in cosmetic products in Nigeria.. Afr J Biotechnol.

[r2] Åkesson AA, Lundh T, Vahter M, Bjellerup P, Lidfeldt J, Nerbrand C (2005). Tubular and glomerular kidney effects in Swedish women with low environmental cadmium exposure.. Environ Health Perspect.

[r3] Al-Saleh I, Al-Enazi S (2011). Trace metals in lipsticks.. Toxicol Environ Chem.

[r4] Al-Saleh I, Al-Enazi S, Shinwari N (2009). Assessment of lead in cosmetic products.. Regul Toxicol Pharmacol.

[r5] ATSDR (Agency for Toxic Substances and Disease Registry). (2008a). Toxicological Profile for Cadmium (Draft for Public Comment).. http://www.atsdr.cdc.gov/toxprofiles/tp.asp?id=48&tid=15.

[r6] ATSDR (Agency for Toxic Substances and Disease Registry). (2008b). Toxicological Profile for Chromium (Draft for Public Comment).. http://www.atsdr.cdc.gov/toxprofiles/tp.asp?id=62&tid=17.

[r7] ATSDR (Agency for Toxic Substances and Disease Registry). (2008c). Toxicological Profile for Manganese (Draft for Public Comment).. http://www.atsdr.cdc.gov/toxprofiles/tp.asp?id=102&tid=23.

[r8] Bach D, Newman AL (2010). Governing lipitor and lipstick: capacity, sequencing, and power in international pharmaceutical and cosmetics regulation.. Rev Int Polit Econ.

[r9] Bouchard M, Laforest F, Vandelac L, Bellinger D, Mergler D (2006). Hair manganese and hyperactive behaviors: pilot study of school-age children exposed through tap water.. Environ Health Perspect.

[r10] Brandao JDO, Okonkwo OJ, Sehkula M, Raseleka RM (2012). Concentrations of lead in cosmetics commonly used in South Africa.. Toxicol Environ Chem.

[r11] Cal/EPA (California Environmental Protection Agency). (2001a). Public Health Goal for Aluminum In Drinking Water.. http://www.oehha.ca.gov/water/phg/pdf/Aluminumf.pdf.

[r12] Cal/EPA (California Environmental Protection Agency). (2001b). Public Health Goals for Chemicals in Drinking Water: Nickel.. http://www.oehha.ca.gov/water/phg/pdf/nickel82001.pdf.

[r13] Cal/EPA (California Environmental Protection Agency). (2006). Public Health Goals for Chemicals in Drinking Water: Cadmium.. http://oehha.ca.gov/water/phg/pdf/122206cadmiumphg.pdf.

[r14] Cal/EPA (California Environmental Protection Agency). (2008a). Appendix D. Individual Acute, 8-Hour, and Chronic Reference Exposure Level Summaries.. http://www.oehha.ca.gov/air/hot_spots/2008/AppendixD1_final.pdf#page=170.

[r15] Cal/EPA (California Environmental Protection Agency). (2008b). Public Health Goals for Chemicals in Drinking Water: Copper.. http://www.oehha.ca.gov/water/phg/pdf/CopperPHG020808.pdf.

[r16] Cal/EPA (California Environmental Protection Agency). (2009). Public Health Goals for Chemicals in Drinking Water: Lead.. http://www.oehha.ca.gov/water/phg/pdf/LeadfinalPHG042409.pdf.

[r17] Cal/EPA (California Environmental Protection Agency). (2011a). Water: All PHGs Developed as of July, 2011.. http://www.oehha.ca.gov/water/phg/allphgs.html.

[r18] Cal/EPA (California Environmental Protection Agency). (2011b). Public Health Goals for Chemicals in Drinking Water: Hexavalent Chromium (Cr VI).. http://www.oehha.ca.gov/water/phg/pdf/Cr6PHG072911.pdf.

[r19] Campaign for Safe Cosmetics. (2007). A Poison Kiss: The Problem of Lead in Lipstick.. http://www.safecosmetics.org/article.php?id=327.

[r20] Centers for Disease Control and Prevention. (2012). Childhood Lead Poisoning and the Environment.. http://ephtracking.cdc.gov/showLeadPoisoningEnv.action.

[r21] Chan OY, Poh SC, Lee HS, Tan KT, Kwok SF (1988). Respiratory function in cadmium battery workers—a follow-up study.. Ann Acad Med Singapore.

[r22] Cook DG, Fahn S, Brait KA (1974). Chronic manganese intoxication.. Arch Neurol.

[r23] Crossgrove J, Zheng W (2004). Manganese toxicity upon overexposure.. NMR Biomed.

[r24] Davison AG, Fayers PM, Taylor AJ, Venables KM, Darbyshire J, Pickering CA (1988). Cadmium fume inhalation and emphysema.. Lancet.

[r25] De Cock M, Maas YGH, Van De Bor M (2012). Does perinatal exposure to endocrine disruptors induce autism spectrum and attention deficit hyperactivity disorders?. Acta Paediatr.

[r26] Dodson RE, Nishioka M, Standley LJ, Perovich LJ, Brody JG, Rudel RA (2012). Endocrine disruptors and asthma-associated chemicals in consumer products.. Environ Health Perspect.

[r27] FDA (Food and Drug Administration). (2005). Guidance for Industry Lead in Candy Likely to be Consumed Frequently by Small Children: Recommended Maximum Level and Enforcement Policy.. http://www.fda.gov/Food/GuidanceRegulation/GuidanceDocumentsRegulatoryInformation/ChemicalContaminantsMetalsNaturalToxinsPesticides/ucm077904.htm.

[r28] FDA (U.S. Food and Drug Administration). (2011). Listing of Color Additives Exempt from Certification. 21CFR73.. http://www.accessdata.fda.gov/scripts/cdrh/cfdocs/cfcfr/CFRSearch.cfm?CFRPart=73&showFR=1&subpartNode=21:1.0.1.1.26.3.

[r29] Ferret P-J, Gomez-Berrada M-P, Galonnier M (2012). Safety evaluation of cosmetic products dedicated to children under 3 years old. Toxicol Lett.

[r30] Gondal MA, Seddigi ZS, Nasr MM, Gondal B (2010). Spectroscopic detection of health hazardous contaminants in lipstick using Laser Induced Breakdown Spectroscopy.. J Hazard Mater.

[r31] Gunduz S, Akman S (2013). Investigation of lead contents in lipsticks by solid sampling high resolution continuum source electrothermal atomic absorption spectrometry.. Regul Toxicol Pharmacol.

[r32] Heisterberg MV (2011). Contact allergy to the 26 specific fragrance ingredients to be declared on cosmetic products in accordance with the EU cosmetics directive.. Contact Dermatitis.

[r33] Hepp NM, Mindak WR, Cheng J (2009). Determination of total lead in lipstick: Development and validation of a microwave-assisted digestion, inductively coupled plasma-mass spectrometric method.. J Cosmet Sci.

[r34] IARC (International Agency for Research on Cancer). (1993). Beryllium, cadmium, mercury, and exposures in the glass manufacturing industry.. IARC Monogr Eval Carcinog Risks Hum.

[r35] Kilburn C (1987). Manganese, malformations and motor disorders: findings in a manganese-exposed population.. Neurotoxicology.

[r36] Kondakis XG, Makris N, Leotsinidis M, Prinou M, Papapetropoulos T (1989). Possible health effects of high manganese concentration in drinking water.. Arch Environ Health.

[r37] Lefebvre M-A, Meuling WJA, Engel R, Coroama MC, Renner G, Pape W (2012). Consumer inhalation exposure to formaldehyde from the use of personal care products/cosmetics.. Regul Toxicol Pharmacol.

[r38] Loretz LJ, Api AM, Barraj LM, Burdick J, Dressler WE, Gettings SD (2005). Exposure data for cosmetic products: lipstick, body lotion, and face cream.. Food Chem Toxicol.

[r39] Moyer B, Nixon ML (2012). Reproductive effects in F1 adult females exposed *in utero* to moderate to high doses of mono-2-ethylhexylphthalate (MEHP).. Reprod Toxicol.

[r40] Nawrot T, Plusquin M, Hogervorst J, Roels H, Celis H, Thijs L (2006). Risk of cancer and environmental exposure to cadmium in a prospective population study.. Lancet Oncol.

[r41] NIOSH (National Institute for Occupational Safety and Health). (2003). NIOSH Manual of Analytical Methods.. http://www.cdc.gov/niosh/docs/2003-154/method-m.html.

[r42] Nogawa K, Tsuritani I, Kido T, Honda R, Ishizaki M, Yamada Y (1990). Serum vitamin D metabolites in cadmium-exposed persons with renal damage.. Int Arch Occup Environ Health.

[r43] Ogoshi K, Moriyama T, Nanzai Y (1989). Decrease in the mechanical strength of bones of rats administered cadmium.. Arch Toxicol.

[r44] Salvador A, Chisvert A. (2007). Part I. General concepts and cosmetic legislation.

[r45] Smith TJ, Petty TL, Reading JC, Lakshminarayan S (1976). Pulmonary effects of chronic exposure to airborne cadmium.. Am Rev Respir Dis.

[r46] Solidum JN, Peji SM. (2011). Lead and cadmium levels of selected beauty products sold in wholesale shops in Manila, Philippines [Abstract]. In: Proceedings from the 5th International Conference on Bioinformatics and Biomedical Engineering, 10–12 May 2011, Wuhan, China.. http://ieeexplore.ieee.org/xpls/abs_all.jsp?arnumber=5781624&tag=1.

[r47] Stayner L, Smith R, Thun M, Schnorr T, Lemen R (1992). A dose-response analysis and quantitative assessment of lung cancer risk and occupational cadmium exposure.. Ann Epidemiol.

[r48] Thun MJ, Schnorr TM, Smith AB, Halperin WE, Lemen RA (1985). Mortality among a cohort of U.S. cadmium production workers—an update.. J Natl Cancer Inst.

[r49] U.S. Environmental Protection Agency. (2012). National Primary Drinking Water Regulations: List of Contaminants & their Maximum Contaminant Level (MCLs).. http://water.epa.gov/drink/contaminants/index.cfm.

[r50] Ulker OC, Ates I, Atak A, Karakaya A (2012). Investigation of sensitizing potency of some cosmetic mixtures. Toxicol Lett.

